# Mapping the current trends and hotspots of extracellular vesicles in Alzheimer's disease: a bibliometric analysis

**DOI:** 10.3389/fnagi.2024.1485750

**Published:** 2024-12-20

**Authors:** Xiaolian Xing, Hongwei Liu, Minheng Zhang, Yang Li

**Affiliations:** ^1^Department of Neurology, First Hospital of Shanxi Medical University, Taiyuan, Shanxi, China; ^2^Department of Neurology, Taiyuan Central Hospital, Taiyuan, Shanxi, China; ^3^Department of Gerontology, The First People's Hospital of Jinzhong, Yuci, Shanxi, China

**Keywords:** Alzheimer's disease, extracellular vesicles, bibliometrics, CiteSpace, VOSviewer

## Abstract

**Background:**

Extracellular vesicles (EVs) have garnered significant attention in Alzheimer's disease (AD) research over the past decade, largely due to their potential in diagnostics and therapeutics. Although the investigation of EVs in AD is a relatively recent endeavor, a comprehensive bibliometric analysis of this rapidly growing field has yet to be conducted.

**Methods:**

This study aims to elucidate and synthesize the relationship between EVs and AD, offering critical insights to guide future research and expand therapeutic possibilities. Over the past 10–15 years, substantial progress has been made in this domain. Through bibliometric techniques, this analysis assesses research performance by examining scientific publications and metrics, including productivity indicators, impact measurements, data mining, and visualization tools.

**Results:**

A total of 602 publications were analyzed using various online platforms for bibliometric analysis. Notably, the number of publications began to increase rapidly in 2018, with China and the United States emerging as leaders in this research area. The National Institute on Aging produced the highest number of publications among institutions. The *Journal of Molecular Sciences* and the *Journal of Biological Chemistry* were the most prolific and most frequently cited journals, respectively. Among individual contributors, Dimitrios Kapogiannis was identified as the most productive author, while Edward J. Goetzl was the most co-cited. The most prevalent keywords included “neurodegenerative diseases,” “exosomes,” “blood biomarkers,” “amyloid beta,” “microglia,” and “tau protein.” Current research hotspots involve microRNA dysregulation, oxidative stress, carboxyl-terminal fragments, small EVs, and mesenchymal stem cell-derived EVs, indicating key areas for future research.

**Conclusion:**

Research on microRNA dysregulation, oxidative stress, carboxyl-terminal fragments, small EVs, and mesenchymal stem cell-derived EVs represents a critical frontier in the study of Alzheimer's disease. The role of EV-mediated neuroinflammation in AD is a focal point of ongoing investigation and will likely shape future developments in the field.

## Introduction

Alzheimer's disease (AD) is among the most prevalent forms of dementia, impacting millions of individuals globally. Both its prevalence and incidence rise with age, contributing to a substantial global burden. Approximately 32 million individuals are affected by AD dementia, 69 million by prodromal AD, and 315 million by preclinical AD. Collectively, these stages represent 416 million people across the AD continuum, comprising roughly 22% of the global population aged 50 and older (Gustavsson et al., 2023). Within the central nervous system (CNS), pathogenic proteins associated with AD, such as tau and amyloid-beta (Aβ) oligomers, have been detected in brain-derived EVsand are implicated in AD pathogenesis (DeLeo and Ikezu, 2018; Guo et al., 2016; Dinkins et al., 2014; Bulloj et al., 2010; Yuyama et al., 2012).

An expanding body of research has established a strong link between EVs and the onset, progression, and potential therapeutic targets for AD. For instance, gene expression analyses have shown that alterations in non-coding RNAs within EVs isolated from post-mortem brain tissue may contribute to AD pathology (Huang Y. et al., 2024). Moreover, viral components contained within EVs have been found to cross the blood-brain barrier (BBB), potentially exacerbating neuroinflammation and dementia. By carrying pro-inflammatory mediators, such as chemokines and cytokines, EVs may connect peripheral infections, such as those caused by herpes simplex virus-1, to AD pathology (Horn and MacLean, 2021). Due to their ability to traverse the BBB, EVs can transmit pro-inflammatory signals from peripheral circulation to the brain, driving chronic neuroinflammation and contributing to AD progression over time (Kodidela et al., 2019; Yates et al., 2019; Anwar and Fathi, 2023).

EVs are secreted by all cell types within the CNS, facilitating communication with neighboring cells or being released into the cerebrospinal fluid (CSF) and bloodstream. These vesicles are produced under both physiological conditions and in response to specific stimuli, transporting molecules that participate in both normal and pathological processes from parent cells to target cells, thereby triggering biological responses (Zaborowski et al., 2015; Panaro et al., 2020). Numerous studies have underscored the dual role of EVs in CNS diseases. On the one hand, EVs can aid in the clearance of toxic proteins and aggregates; on the other hand, they can deliver toxic cargo to healthy cells, thereby promoting disease progression (Graykowski et al., 2020). Recent research has demonstrated a significant correlation between the cargo of plasma neuronal-derived EVs and Aβ deposition in patients across the Alzheimer's continuum (Li et al., 2022). Additionally, microglia have been shown to facilitate tau propagation via EV secretion, with reductions in EV synthesis leading to a marked decrease in tau spread (Asai et al., 2015).

Given the increasing body of research on EVs in AD, a bibliometric analysis can deliver a detailed and systematic overview of emerging trends, offering valuable insights for both new and experienced researchers. Although many studies have explored the role of EVs in AD, quantitative analyses of the literature remain scarce. To fill this gap, we employed bibliometric techniques to evaluate the existing research, identify key hotspots and emerging trends, and synthesize prominent themes highlighted in reviews.

## Methods

### Data sources and search strategy

**Figure 1:**
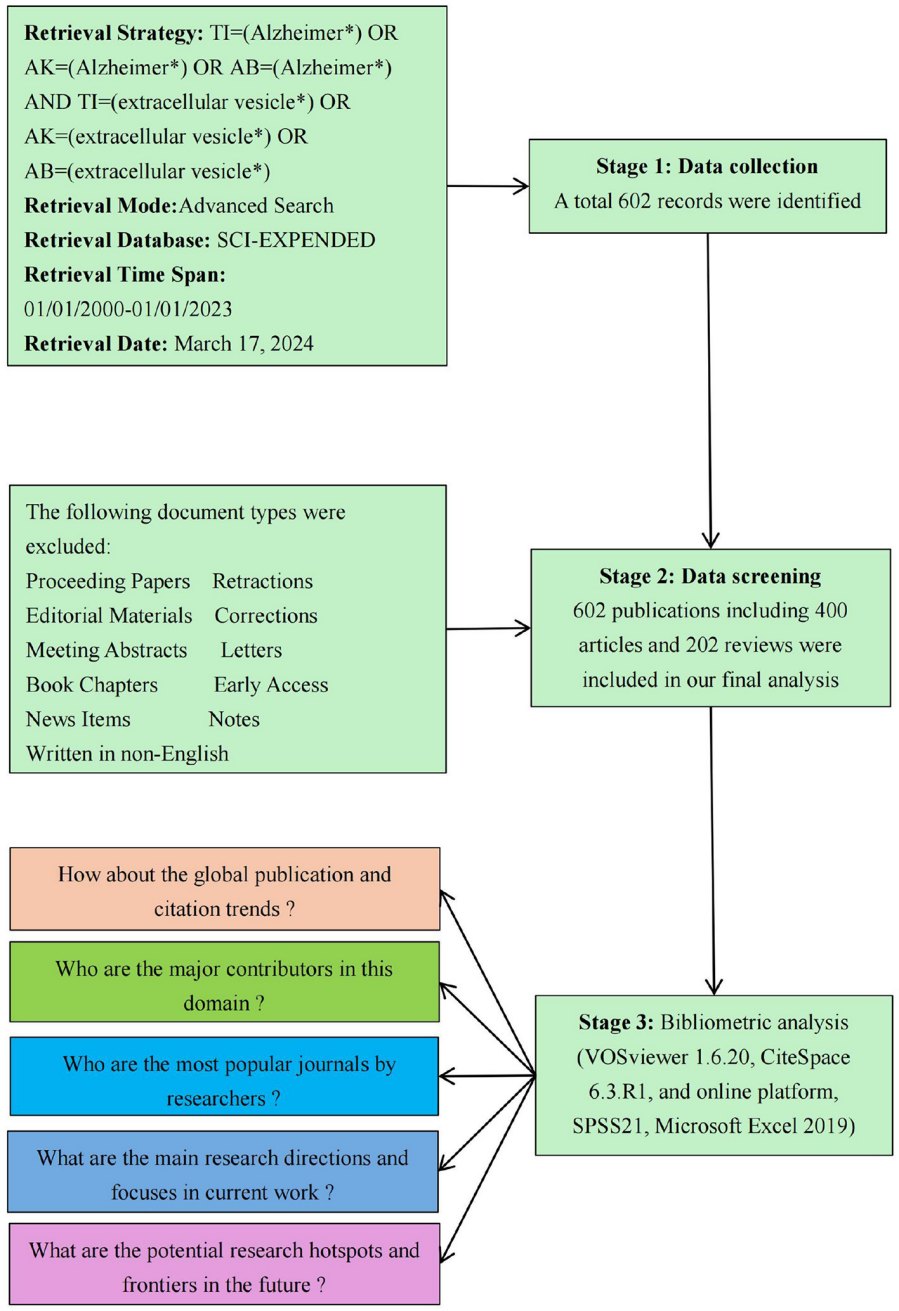
Flowchart illustrating the study identification and selection process.

The ethical board of Taiyuan Central Hospital granted a waiver of informed consent for this study, as it involved only data analysis and did not include any identifiable personal information. The data for this study were drawn from the Web of Science Core Collection (WoSCC) database, an esteemed resource known for its expansive coverage of more than 9,000 scholarly journals. This database serves as an indispensable tool for bibliometric analyses across diverse research domains. The study specifically employed the Science Citation Index Expanded (SCI Expanded) as the central index. The search strategy was formulated as follows: TI=(Alzheimer^*^) OR AK=(Alzheimer^*^) OR AB=(Alzheimer^*^) AND TI=(extracellular vesicle^*^) OR AK=(extracellular vesicle^*^) OR AB=(extracellular vesicle^*^). The search strategy focused on identifying publications released between January 1, 2001, and December 31, 2023, limiting the scope to Articles and Review Articles written in English. To ensure the robustness of the dataset, two experts, each with over 5 years of experience in systematic reviews and scientometric analysis, conducted a meticulous title screening of all retrieved articles. Any discrepancies during the selection process were resolved through consultation with a third author. This rigorous selection methodology resulted in a final dataset comprising 602 entries, including 400 original research articles and 202 review articles. The retrieval process is illustrated in Figure 1. All selected records were subsequently exported in TXT format for further analysis.

### Data analysis

For data analysis, we utilized VOSviewer (version 1.6.20), a highly regarded bibliometric software renowned for its ability to extract and visualize key insights from extensive datasets of academic publications (Yeung and Mozos, 2020; van Eck and Waltman, 2010; Pan et al., 2018). In this study, VOSviewer was pivotal in conducting various analyses, including country and institution analysis, journal and co-cited journal analysis, author and co-cited author analysis, and keyword co-occurrence analysis. The visual maps generated by VOSviewer represent entities such as countries, institutions, journals, and authors as nodes. The size and color of each node indicate the volume and classification of these entities, while the thickness of the connecting lines illustrates the degree of collaboration or co-citation (Wu et al., 2021; Zhang et al., 2020). In addition, we employed CiteSpace (version 6.3.R1), a bibliometric tool widely recognized for its ability to identify research hotspots and trace the evolution of specific academic fields, thus offering valuable insights for future research directions (Synnestvedt et al., 2005). CiteSpace excels at detecting citation bursts and keyword bursts, which are instrumental in identifying emerging research trends. Moreover, the software provides a wide array of visual analysis tools, such as clustering publication data and generating keyword timeline graphs, thereby offering a comprehensive overview of the historical and current developments within a given field (Han et al., 2023).

## Results

### Annual publications and citation trends

Bibliometrics has become a fundamental tool in the biomedical field, playing key roles in disease diagnosis, guiding treatment strategies, and identifying emerging research trends. This study explores the evolving landscape of EVs in AD from 2001 to 2023. A significant increase in scholarly publications has been observed over this period, with the United States leading in contributions. Despite this growth, collaboration among authors and institutions presents complex challenges, reflecting the intricate network of scientific cooperation in this field.

**Figure 2:**
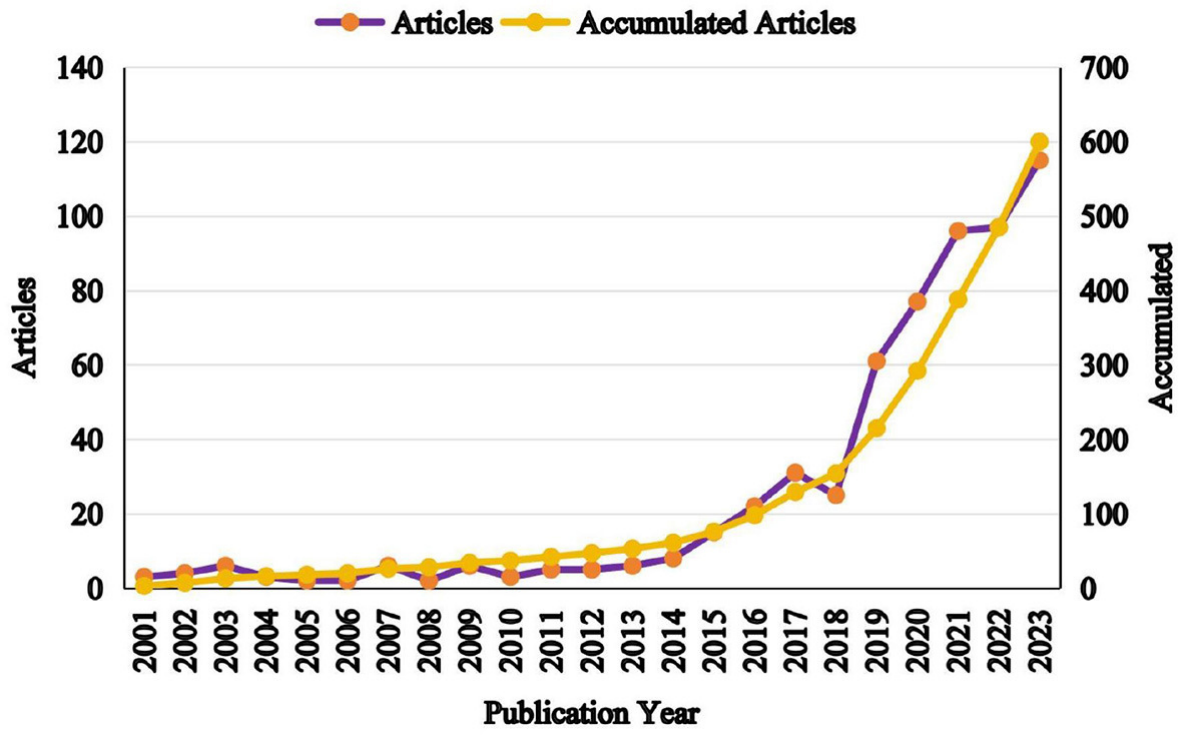
Annual number of publications and cumulative publication count.

As illustrated in Figure 2, the publication trends from 2001 to 2023 can be divided into two distinct phases: an initial slow-growth period from 2001 to 2018, followed by a rapid growth phase from 2018 to 2023. During the first phase, only 45 articles were published, accounting for 7.5% of the total output, with slight declines observed during the periods of 2003–2006, 2009–2012, and 2017–2018. In contrast, the second phase saw a sharp rise in productivity, culminating in 524 publications with a 3.6-fold increase compared to the earlier period. This surge can be attributed to the growing recognition of the diagnostic and therapeutic potential of peripheral EVs in AD.

### Distribution of countries/regions

The top ten contributing countries in this field include the United States, leading with 229 publications, followed by China with 98, Japan with 30, and South Korea with 29 in Asia. In Europe, Italy contributed 57 publications, Germany 49, Spain 35, England 28, and Sweden 24. Australia also made a significant contribution with 26 publications in Oceania (Table 1). Collectively, the United States and China contribute to more than half of the total publications, underscoring their substantial influence in this area of research.

**Table 1:** Top 10 most publication countries/regions related to extracellular vesicles in Alzheimer's disease.

**Rank**	**Institutions**	**Total link strength**	**Total citations**	**Count (%)**
1	United States	109	11,417	229 (29.47%)
2	China	25	2,251	98 (12.61%)
3	Italy	32	1,985	57 (7.33%)
4	Germany	43	3,485	49 (6.30%)
5	Spain	30	1,110	35 (4.50%)
6	Japan	14	1,144	30 (3.86%)
7	South Korea	14	971	29 (3.73%)
8	England	34	975	28 (3.60%)
9	Australia	20	2,173	26 (3.35%)
10	Sweden	19	1,579	24 (3.09%)

**Figure 3:**
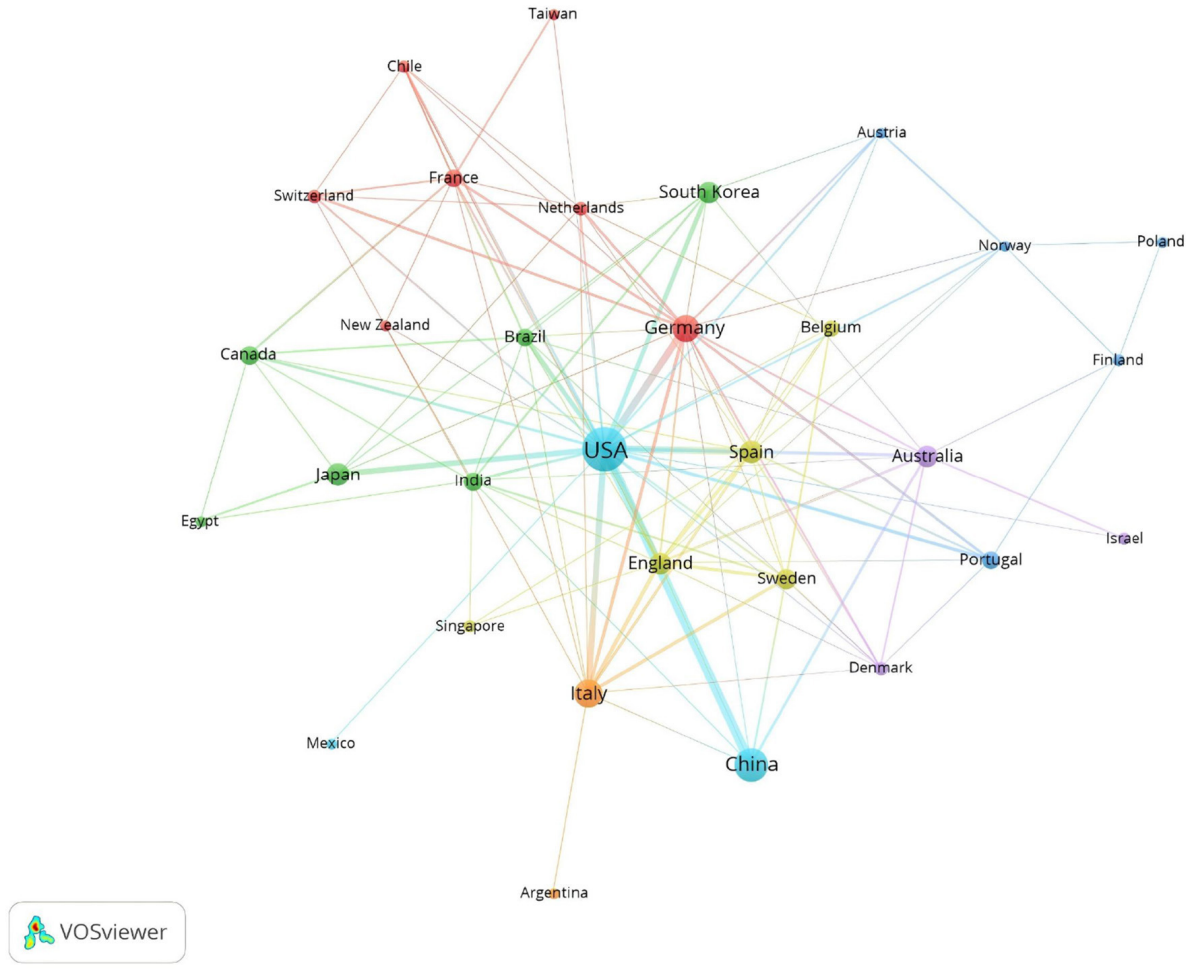
Geographic distribution of countries/regions and their collaboration network.

In terms of citations, the United States leads with 11,417 citations, followed by Germany with 3,485, China with 2,251, Australia with 2,173, and Italy with 1,985 (Table 1). Figure 3 visualizes this global distribution, positioning the United States as the leader in both publication volume and citations. Additionally, the United States demonstrates the most extensive network of collaborations, emphasizing the importance of enhancing international cooperation to further advance the field.

### Analysis of institution publications

A total of 117 institutions have collectively contributed over 600 studies on EVs in AD from 2001 to 2023. Table 2 highlights the top 10 institutions based on their publication output during this period. Leading the list is the National Institute on Aging (NIA) with 27 publications, followed by Boston University with 20, the University of California, San Diego with 16, and both the University of California, San Francisco and Harvard Medical School with 14 publications each. Notably, the NIA also exhibits the highest overall collaboration strength, underscoring the prominent role of U.S. institutions in advancing research in this area.

**Table 2:** Top 10 most publication institutions related to extracellular vesicles in Alzheimer's disease.

**Rank**	**Institutions**	**Total link strength**	**Count (%)**
1	National Institute on Aging	34	27 (4.45%)
2	Boston University	25	20 (3.29%)
3	University of California, San Diego	31	16 (2.64%)
4	University of California, San Francisco	22	14 (2.31%)
5	Harvard Medical School	29	14 (2.31%)
6	La Trobe University	14	13 (2.14%)
7	The University of Melbourne	8	12 (1.98%)
8	Johns Hopkins University	14	12 (1.98%)
9	CNR	20	12 (1.98%)
10	Columbia University	16	10 (1.65%)

### Contribution of journals

**Figure 4:**
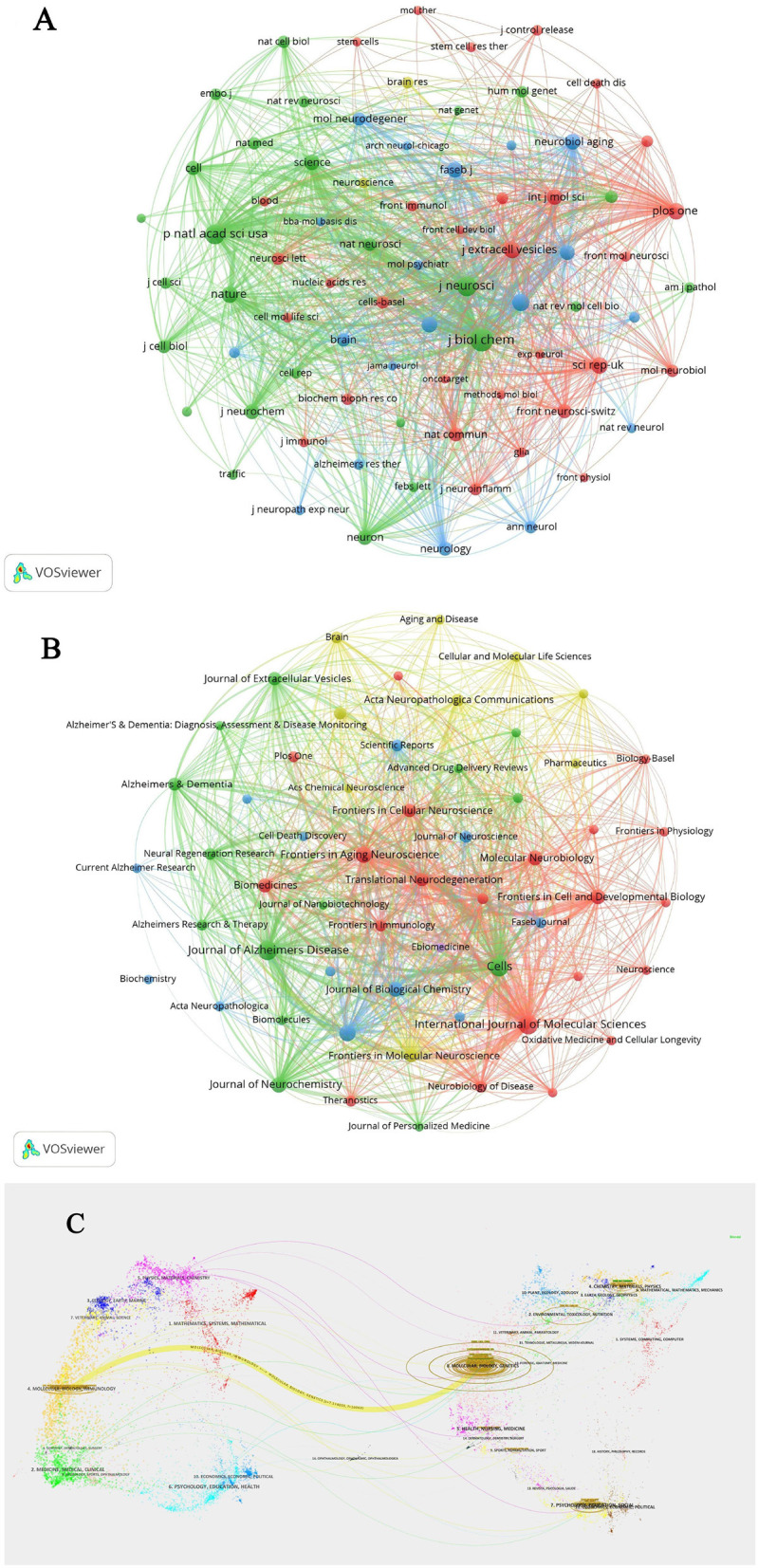
**(A)** Clustering analysis of journals related to extracellular vesicles in Alzheimer's disease. **(B)** Co-cited journals clustering analysis of extracellular vesicles in Alzheimer's disease. **(C)** Dual-map overlay of journal-published research.

Over the past two decades, more than 600 articles on EVs in AD have been published across 57 journals. The top 10 journals account for 165 of these publications, representing 17.58% of the total output (Table 3). The *International Journal of Molecular Sciences* (IF: 5.6, Q1) leads with 27 articles. Most of these journals are ranked in Q1 or Q2 according to JCR 2022 standards, with *Cells* (IF: 6.0, Q2) having the highest impact factor, contributing 24 articles and 518 citations. The *Journal of Biological Chemistry* (IF: 4.8, Q2) stands out with 1,224 citations, making it the most cited journal in this field, followed by the *Journal of Alzheimer's Disease* with 1,020 citations and the *International Journal of Molecular Sciences* with 909 citations. These journals have exerted substantial influence within the research community, though there remains potential for deeper exploration in this evolving field. The clustering analysis of journals and co-cited journals is illustrated in Figures 4A, B. Through VOSviewer software, we can visually examine the collaboration network between these journals, offering a more nuanced perspective on their cooperative relationships. The periodicals are organized into four clusters according to co-citation frequency, suggesting that articles published within the same journal often share common research directions or underlying conceptual frameworks. The Proceedings Of The National Academy Of Sciences Of The United States Of America (Proc Natl Acad Sci USA), and the Journal of Biological Chemistry (J Biol Chem) exhibit high co-citation frequencies and exert substantial influence within the field. In the dual-map overlay of journal-published research (Figure 4C), a prominent citation pathway, highlighted in yellow, stands out. This colored path illustrates the citation relationships across various fields, showing that studies published in journals centered on molecular, biological, and genetic research predominantly cite work from journals specializing in molecular, biological, and immunological research. This pattern underscores the interdependence and interconnectedness of diverse disciplines, collectively advancing our understanding of AD.

**Table 3:** Top 10 journals and co-cited journals related to extracellular vesicles in Alzheimer's disease.

**Rank**	**Journal**	**Count (%)**	**Total citations**	**IF (2022)**	**JCR quantile**	**Co-cited-journal**	**Citation**	**IF (2022)**	**JCR quatile**
1	INT J MOL SCI	27 (7.07%)	909	5.6	Q1	J Biol Chem	2,058	4.8	Q2
2	J Alzheimers Dis	25 (6.54%)	1,020	4.0	Q2	Proc Natl Acad Sci USA	1,427	11.1	Q1
3	Cells	24 (6.28%)	518	6.0	Q2	J Neurosci	1,418	5.3	Q1
4	Front Aging Neurosci	17 (4.45%)	792	4.8	Q2	J Alzheimers Dis	998	4.0	Q2
5	Frontiers in Neuroscience	16 (4.19%)	897	4.3	Q2	Nature	882	64.8	Q1
6	Front Neurosci	13 (3.40%)	868	4.7	Q2	Plos One	878	3.7	Q2
7	Mol Neurobiol	12 (3.14%)	301	5.1	Q2	J Extracell Vesicles	838	16.0	Q1
8	J Biol Chem	12 (3.14%)	1,224	4.8	Q2	Scientific Reports	774	4.6	Q2
9	Front Cell Dev Biol	12 (3.14%)	258	5.5	Q1	INT J MOL SCI	764	5.6	Q1
10	Biomedicines	10 (2.62%)	82	4.7	Q1	Science	745	56.9	Q1

### Authors and co-cited authors

**Figure 5:**
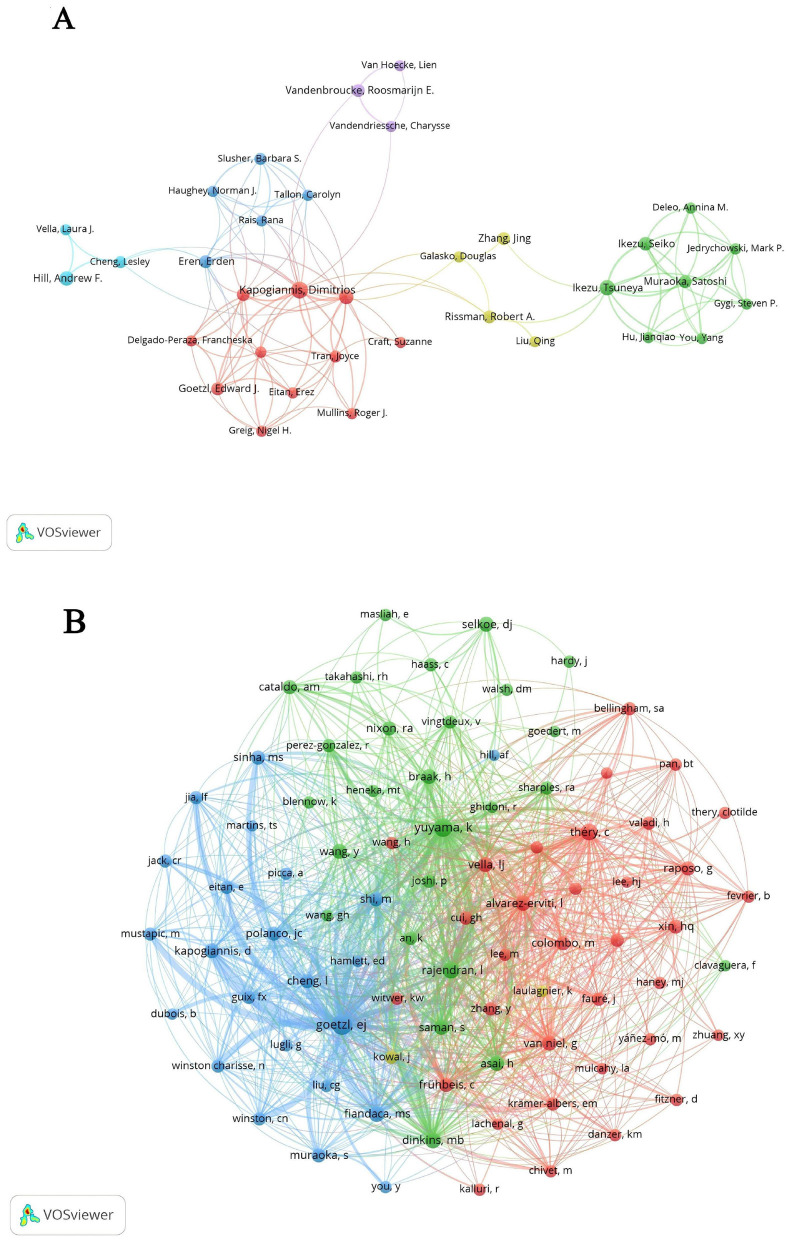
**(A)** Co-authorship network related to extracellular vesicles in Alzheimer's disease. **(B)** Co-cited authors network analysis.

Table 4 provides an overview of the top 10 authors by publication count and citation frequency, shedding light on the key figures in EV and AD research. Kapogiannis D leads the field with 27 publications, followed by Ikezu T, contributing 7.69% of the total publications, and Hill AF with 5.56%. Notably, Kapogiannis D, Ikezu T, and Mustapic M exhibit strong collaborative interactions, as evidenced by their high total link strengths. Figure 5A illustrates the collaborative network of authors in EVs and AD research, highlighting distinct clusters that represent focused areas of study and demonstrate the interdisciplinary nature of this field. The cluster closely associated with Kapogiannis D's team primarily focuses on neuroinflammation and its relationship to EVs, showcasing collaborations that bridge neuroimmunology and molecular biology. Meanwhile, the cluster linked to Ikezu T's team emphasizes the therapeutic potential of EVs, particularly in preclinical and clinical applications. These co-authorship links between Kapogiannis D and Ikezu T underscore the integration of basic EVs biology with its clinical implications, facilitating the development of diagnostic biomarkers and innovative therapeutic approaches. In terms of co-citations, Goetzl EJ stands out with 426 citations and is among seven authors who have garnered over 200 citations each, indicating their significant impact within the scholarly community (Table 4). Figure 5B depicts the co-cited authors' collaboration network, divided into four distinct clusters, underscoring the dynamic and active collaborations among scholars such as Goetzl EJ, Yuyama K, and Rajendran L. This visualization highlights the interconnected nature of research in this rapidly evolving field.

**Table 4:** Top 10 authors and co-cited authors related to extracellular vesicles in Alzheimer's disease.

**Rank**	**Author**	**Count (%)**	**Total link strength**	**Co-cited author**	**Citation**	**Total link strength**
1	Kapogiannis D	27 (11.54%)	69	Goetzl EJ	426	9,277
2	Ikezu T	18 (7.69%)	46	Yuyama K	295	6,850
3	Hill AF	13 (5.56%)	9	Rajendran L	205	4,073
4	Mustapic M	13 (5.56%)	41	Théry C	179	3,036
5	Ikezu S	11 (4.70%)	34	Dinkins MB	150	3,600
6	Rissman RA	9 (3.85%)	8	Fiandaca MS	147	3,137
7	Goetzl EJ	8 (3.42%)	24	Alvarez-Erviti L	127	3,010
8	Muraoka S	8 (3.42%)	32	Saman S	126	2,981
9	Vandenbroucke RE	8 (3.42%)	9	Kapogiannis D	122	2,578
10	Eren E	7 (2.99%)	22	van Niel G	122	2,702

### References and co-cited references

**Figure 6:**
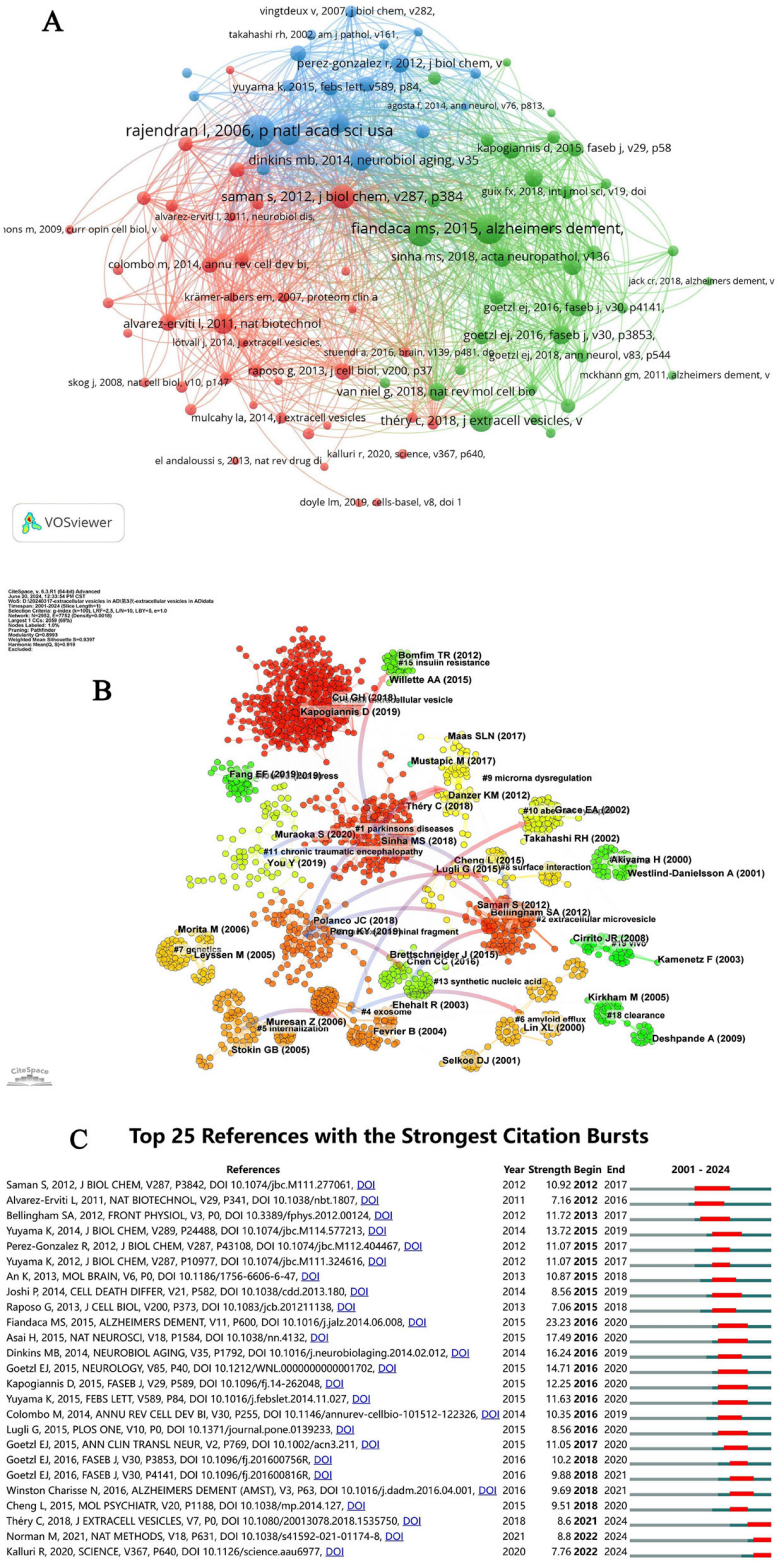
**(A)** Network diagram of document relationships generated by VOSviewer. **(B)** Cluster view of references on extracellular vesicles in Alzheimer's disease. **(C)** CiteSpace visualization map of the top 25 references with the strongest citation bursts in extracellular vesicles research on Alzheimer's disease.

Table 5 lists the top 10 most-cited articles in the field, collectively accounting for 22.40% of total citations. Noteworthy is the 2016 publication by Rajendran L, which amassed 158 citations. Among these top 10 articles, five originated from U.S. institutions and two from Japanese institutions, underscoring the global contributions to pivotal research in this area. The works of Rajendran L and Fiandaca MS have been disseminated through international collaborations, emphasizing the critical role of highly cited articles in shaping research directions in this field. A co-citation analysis of the literature on EVs in AD was conducted using CiteSpace, as illustrated in Figure 6A. This analysis identified 19 co-reference clusters (Figure 6B), revealing that highly cited articles significantly contribute to the influential research corpus. Cluster #0 emerged as the largest, indicating a high frequency of citations to associated papers. Keywords such as “small extracellular vesicles” appeared most frequently, followed by “Parkinson's disease” in Cluster #1, “extracellular microvesicles” in Cluster #2, and “carboxyl-terminal fragment” in Cluster #3. The visual data from Figure 6A also highlighted a discernible shift in research focus toward “oxidative stress,” “carboxyl-terminal fragment,” “mesenchymal stem cell-derived EVs,” and “microRNA dysregulation” in recent years, indicating potential shifts in research paradigms. Figure 6C catalogs the top 25 references that have experienced significant citation bursts related to EVs in AD, offering valuable insights into evolving research hotspots. A closer examination of these articles reveals critical trends in the field. Notably, the 2015 study by Fiandaca MS et al., “Identification of preclinical Alzheimer's disease by a profile of pathogenic proteins in neurally derived blood exosomes: A case-control study,” published in Alzheimer's and Dementia, achieved the strongest citation burst (23.23) from 2016 to 2020. This study underscores the pivotal role of biomarkers from extracellular vesicles in early AD detection, marking significant trends within the research landscape (Fiandaca et al., 2015).

**Table 5:** The top 10 most cited research papers.

**Rank**	**Author**	**Journal**	**Year**	**Citations**	**Descriptions**
1	Asai H	Nat Neurosci	2015	771	Microglia and exosomes contribute to the progression of tauopathy and that the exosome secretion pathway may be a therapeutic target.
2	Simons K.	Proc Natl Acad Sci USA	2006	158	Exosomal proteins were found to accumulate in the plaques of AD patient brains, suggesting a role in the pathogenesis of AD.
3	Fiandaca MS	Alzheimers Dement	2015	143	Levels of P-S396-tau, P-T181-tau, and Aβ1-42 in extracts of neurally derived blood exosomes predict the development of AD up to 10 years before clinical onset.
4	Saman S	J Biol Chem	2012	109	Exosome-mediated secretion of phosphorylated tau may play a significant role in the abnormal processing of tau and in the genesis of elevated CSF tau in early AD.
5	Théry C	J Extracell Vesicles	2018	102	The MISEV2018 guidelines include tables and outlines of suggested protocols and steps to follow to document specific EV-associated functional activities. Finally, a checklist is provided with summaries of key points.
6	Igarashi Y	J Biol Chem	2012	95	A novel mechanism responsible for clearance of Aβ through its association with exosomes. The modulation of the vesicle release and/or elimination may alter the risk of AD.
7	Dinkins MB	Neurobiol Aging	2014	92	GW4869 reduces amyloid plaque formation *in vivo* by preventing exosome secretion and identifies nSMase2 as a potential drug target in AD by interfering with exosome secretion.
8	Wood MJ	Nat Biotechnol	2011	85	The therapeutic potential of exosome-mediated siRNA delivery was demonstrated by the strong mRNA (60%) and protein (62%) knockdown of BACE1, a therapeutic target in Alzheimer's disease, in wild-type mice.
9	Sardar Sinha M	Acta Neuropathol	2018	83	Exosomes are centrally involved in Alzheimer's disease and that they could serve as targets for development of new diagnostic and therapeutic principles.
10	Yuyama K	J Biol Chem	2014	83	Intracerebrally administered exosomes can act as potent scavengers for Aβ by carrying it on the exosome surface GSLs and suggest a role of exosomes in Aβ clearance in the central nervous system. Improving Aβ clearance by exosome administration would provide a novel therapeutic intervention for Alzheimer disease.

### Keyword analysis of global research

#### Keyword timeline analysis

**Figure 7:**
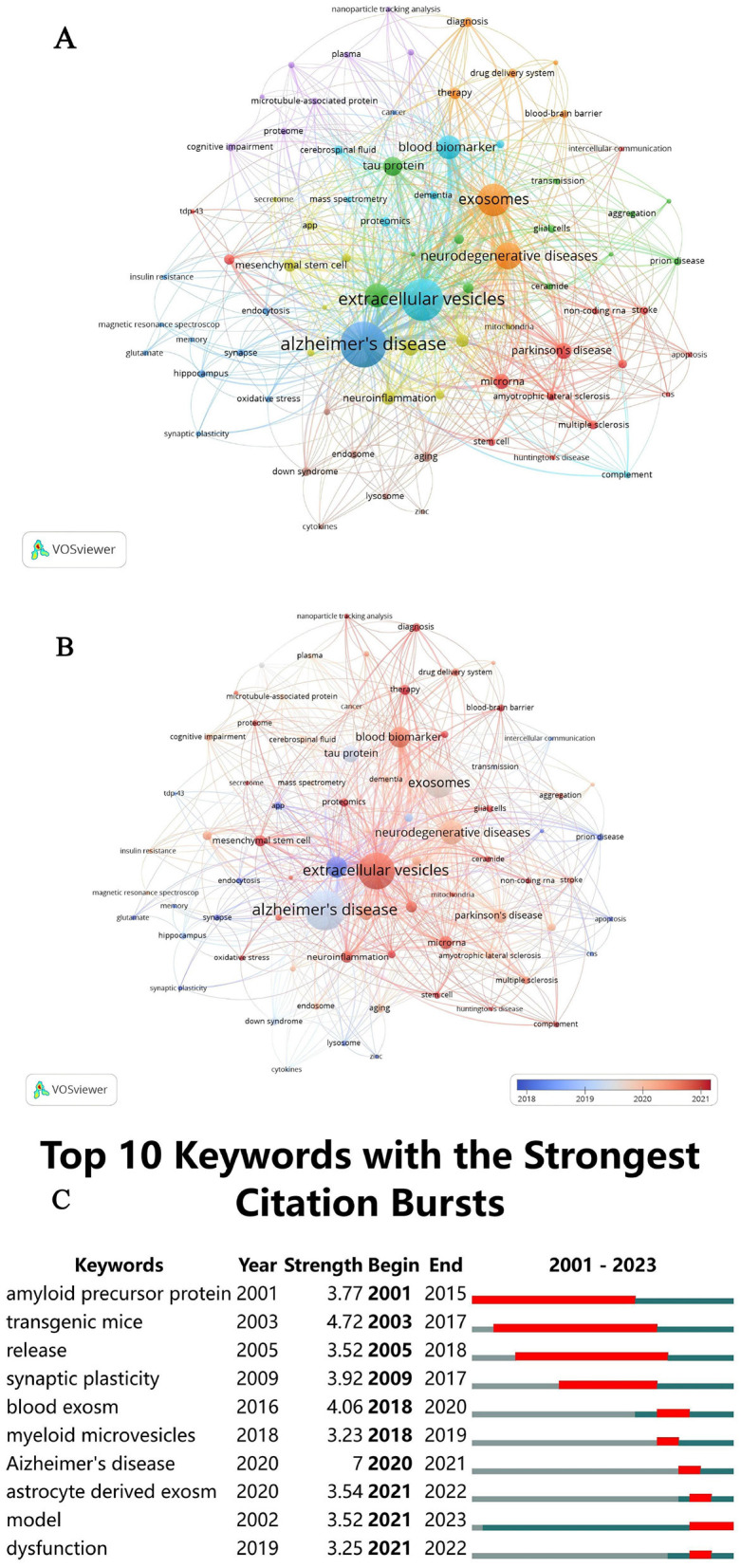
**(A)** Keyword co-occurrence network clustering diagram. **(B)** Network-view map of keyword co-occurrence. **(C)** CiteSpace visualization map of the top 10 keywords with the strongest citation bursts.

The keyword co-occurrence and clustering analysis, shown in Figure 7A, has systematically identified eight distinct clusters, each representing a focal research area within AD. This strategic grouping of keywords into clusters provides a clear visualization of prevalent research hotspots and emerging frontiers, essential for guiding future academic investigations. Red Cluster: Focused on “microglia,” “Parkinson's disease,” and “multiple sclerosis,” this cluster highlights a strong interest in neuroinflammatory processes that overlap with various neurodegenerative disorders. Orange Cluster: Including “exosomes,” “neurodegenerative diseases,” and “tau protein,” this cluster underscores significant research into molecular pathways and the identification of potential biomarkers specific to neurodegenerative conditions. Blue Cluster: Comprising “blood markers,” “extracellular vesicles,” and “Alzheimer's disease,” this cluster signals a robust focus on diagnostic markers and the systemic dimensions of AD. Additionally, keywords such as “amyloid-beta,” “neuroinflammation,” and “astrocyte” appear across multiple clusters, reflecting their pivotal roles in AD research. This comprehensive analysis not only clarifies the primary focus areas within AD but also indicates shifting dynamics within the field. Notably, there is a growing emphasis on the role of EVs in dementia progression, the impact of inflammatory processes, and the broader spectrum of neurodegeneration. These insights are crucial for researchers seeking to direct their efforts toward the most pressing and promising domains in AD research, potentially accelerating advancements in understanding and treating this complex condition.

#### Keywords timeline viewer

Figure 7B provides a detailed analysis of the mean publication year for each keyword, utilizing a color gradient from blue to red to illustrate temporal progression. The data show that key terms such as “Alzheimer's disease,” “extracellular vesicles,” “exosomes,” “blood markers,” “neurodegenerative diseases,” “amyloid-beta,” “tau protein,” “microglia,” and “neuroinflammation” were predominantly used between 2018 and 2021. Specific peaks include “amyloid-beta” in 2018, “Alzheimer's disease,” “tau protein,” and “exosomes” in 2019, followed by “neuroinflammation,” “neurodegenerative diseases,” “blood markers,” “astrocyte,” “microglia,” and “extracellular vesicles” in 2020 and 2021. Recently, “extracellular vesicles” and “microglia” have emerged as focal points, particularly over the past 2 years. These trends highlight significant shifts in research focus, enriching our understanding of the evolving research landscape and informing future directions.

### Citation bursts analysis

The examination of keyword frequency provides insight into the primary research directions, emphasizing the areas that are currently receiving the most attention. As presented in Table 6, the top 10 most frequently occurring keywords in the literature on EVs in AD are “neurodegenerative diseases” (110), “exosomes” (162), “blood biomarker” (88), “amyloid beta” (67), “tau protein” (55), “Parkinson's disease” (37), “microRNA” (36), “microglia” (34), “neuroinflammation” (26), and “mesenchymal stem cell” (24). These keywords underscore pivotal areas of research, illustrating both well-established priorities and emerging avenues in the exploration of extracellular vesicles in the context of Alzheimer's disease.

**Table 6:** Top 10 keywords related to extracellular vesicles in Alzheimer's disease.

**Rank**	**Keyword**	**Occurrences**	**Total link strength**	**Average citation**
1	Neurodegenerative diseases	110	377	44
2	Exosomes	162	559	61
3	Blood biomarker	88	297	38
4	Amyloid beta	67	200	73
5	Tau protein	55	196	48
6	Parkinson's disease	37	160	47
7	MicroRNA	36	128	32
8	Microglia	34	125	66
9	Neuroinflammation	26	88	40
10	Mesenchymal stem cell	24	79	13

The concept of “outbreak keywords” identifies the most dynamic research areas within the field. Keywords that have maintained prominence during their outbreak periods in recent years indicate ongoing, cutting-edge research efforts. As illustrated in Figure 7C, the top 10 keywords with the highest outbreak intensity include “amyloid precursor protein,” “transgenic mice,” “release,” “synaptic plasticity,” “blood exosome,” “myeloid microvesicles,” “Alzheimer's disease,” “astrocyte-derived exosome,” “model,” and “dysfunction.” The sustained prominence of these keywords through 2021 highlights the continuing emphasis on pivotal research areas concerning EVs in AD. These keywords collectively reflect major mechanistic themes, including protein aggregation (e.g., amyloid precursor protein and tau), key cellular signaling pathways such as synaptic plasticity, neuroinflammation, and neuronal communication, as well as the role of EVs-mediated cellular communication, with a particular focus on the involvement of myeloid microvesicles and astrocyte-derived exosomes in neuroinflammation and neurodegeneration. The recurring appearance of keywords such as “model” and “dysfunction” further indicates a growing focus on the development of *in vivo* models to investigate the molecular mechanisms underlying EVs-mediated pathology and the dysfunctional pathways by which EVs contribute to disease progression. This underscores the increasing recognition of EVs as pivotal contributors to the pathophysiology of AD, presenting opportunities for novel biomarkers and therapeutic targets in future clinical interventions.

## Discussion

This study explored the dynamic research trends and key focus areas within the field of EVs in AD. A thorough analysis was conducted on 602 articles published between 2000 and 2023, sourced from the Web of Science database. Employing advanced bibliometric tools, including VOSviewer (version 1.6.20) and CiteSpace (version 6.3.R1), we systematically examined the evolving landscape and developmental trajectories within this research domain.

### General information

The examination of annual publication volumes provides crucial insights into the trajectory of research on EVs in AD (Yuan W. C. et al., 2023). Notably, from 2018 to 2023, there has been a substantial increase in publications, with over three-quarters (78.23%) of the total output occurring within the last 5 years, as illustrated in Figure 2. This marked surge underscores the growing academic interest in the intersection of EVs and AD. A geographical analysis of the research landscape reveals that the United States not only leads in the number of published articles but also in accrued citations, reaffirming its dominant role in the global research arena. Together, the United States and China contribute 56.05% of the total literature on EVs in AD, with respective shares of 29.47 and 12.61%. This dominant contribution highlights their leadership in this specialized field. Moreover, the presence of seven of the top 10 publishing institutions within the United States further reinforces its influential role in advancing research in this area.

As detailed in Table 3, in terms of publication impact, the *International Journal of Molecular Sciences* stands out with the highest number of studies related to EVs in AD and is also the ninth most cited journal. Notably, four of the top 10 journals with the most publications also rank among the top 10 co-cited journals, including the *Journal of Alzheimer's Disease*, the *Journal of Biological Chemistry*, and the *International Journal of Molecular Sciences*. This overlap indicates not only their substantial influence within the research community but also the high quality of studies published therein, as all top 10 journals are classified as either Q1 or Q2. The scope of these journals spans various disciplines, from neurosciences to biochemistry, molecular biology, and other interdisciplinary fields, as reflected by their co-citation frequencies. This diversity is further corroborated by the dual-map overlay analysis, which reveals a broad yet interconnected research ecosystem.

### Hotspots and frontiers

Keywords and references are foundational elements in scholarly literature, illuminating the core research themes within a field. Reference clusters and citation bursts are particularly valuable for uncovering emerging topics and understanding which areas are gaining traction within the discipline. These analytical tools are essential for detecting shifts in research focus and guiding scholars toward areas most likely to influence future studies (Chen and Song, 2019; Ma et al., 2022). Among the top 10 co-cited references on AD, four significant studies focus on amyloid plaque formation, a critical marker of AD pathology. One pivotal study reveals the accumulation of exosomal proteins within amyloid plaques in AD patient brains, suggesting that exosomes play a significant role in plaque composition (Rajendran et al., 2006). Another investigation demonstrates that levels of phosphorylated tau proteins (P-S396-tau and P-tau-181) and Aβ-42 in neurally derived blood EVs can predict the onset of AD up to 10 years before clinical symptoms emerge (Fiandaca et al., 2015). A third study introduces a novel mechanism for Aβ clearance through its association with EVs, potentially opening new therapeutic pathways (Yuyama et al., 2012). The fourth study shows that inhibiting exosome secretion with GW4869 can reduce amyloid plaque formation *in vivo*, identifying nSMase2 as a potential drug target (Dinkins et al., 2014). Additionally, another study explores the therapeutic potential of intracerebrally administered exosomes as Aβ scavengers, binding Aβ to the exosome surface through glycosphingolipids, thus highlighting a possible method for Aβ clearance in the central nervous system (Yuyama et al., 2014). Current citation bursts, as depicted in Figure 6C, point to five references that merit further exploration, particularly those addressing the impact of EVs on tauopathy progression in AD. In addition to amyloid-related studies, tau pathology has emerged as a critical theme in the co-cited references. Multiple studies underscore the role of EVs in facilitating tau propagation across neural networks, thereby contributing to the spread of tau pathology in AD (Asai et al., 2015; Polanco et al., 2018). For example, research has shown that microglia-derived EVs transport phosphorylated tau proteins, promoting their accumulation in recipient neurons and accelerating the spread of tau-related damage (Sinha et al., 2018; Trotta et al., 2018). Another pivotal study demonstrated that inhibiting EVs production in AD models significantly curtailed the propagation of tau, highlighting the therapeutic potential of targeting EVs pathways (Garcia-Contreras and Thakor, 2023). Moreover, EVs have been implicated in transporting tau protein fragments, which can seed tau aggregation in distant brain regions, further exemplifying the role of EVs in the intercellular dissemination of pathological tau (Polanco et al., 2018; Pérez et al., 2019). This growing body of evidence underscores the increasing recognition of EVs as key mediators in both amyloid and tau pathology in AD, presenting opportunities for the development of targeted therapies (Asai et al., 2015; Trotta et al., 2018). Notably, as shown in Figure 6B, the earliest and largest reference cluster, Cluster #0, focuses on small extracellular vesicles. This prominent cluster reflects the early recognition of sEVs as key players in AD and neurodegenerative research. The central role of sEVs in transporting amyloid-beta, tau, and other proteins implicated in disease pathogenesis has garnered considerable attention. Furthermore, the sustained focus on small EVs suggests their potential as biomarkers for early detection and targets for therapeutic intervention in AD. The size and longevity of Cluster #0 underscore the growing consensus in the scientific community regarding the importance of sEVs in disease progression and their therapeutic promise.

Table 5 highlights several high-frequency keywords beyond “Alzheimer's disease” and “extracellular vesicles,” including “neuroinflammation,” “neurodegenerative diseases,” “blood biomarker,” “amyloid beta,” “tau protein,” and “microglia.” These terms represent critical research areas at the intersection of inflammation, immunology, and neuroscience, encompassing themes such as oxidative stress, cytokine interactions, phagocytosis, neurodegeneration, and neuroprotection.Recent prominent keywords identified in Figure 7B, such as “extracellular vesicles,” “blood biomarker,” “mesenchymal stem cell,” “neuroinflammation,” “proteomics,” “microRNA,” and “astrocyte” reflect the forefront of research and potential future directions in the field. These terms emphasize ongoing efforts to unravel the pathogenesis of AD and explore novel therapeutic strategies, marking them as pivotal areas for future investigation.

### The effect of EVs-mediated neuroinfammation on AD progression

Based on analyses of keyword occurrences, chronological trends, reference clustering, and the top 10 co-cited references, neuroinflammation remains a central focus and a critical frontier in neuroscientific research. Notably, some of the earliest and most impactful studies in this domain have concentrated on small extracellular vesicles (sEVs), as depicted in Figure 6B. This foundational work has paved the way for deeper exploration of the mechanisms and implications of neuroinflammatory processes, underscoring its enduring relevance and potential to advance the field. sEVs are nanosized vesicles, typically < 200 nm, characterized by a bilayered membrane structure. These vesicles play a crucial role in intercellular communication by transporting bioactive molecules to recipient cells (Théry et al., 2018; Cocozza et al., 2020). A significant body of research utilizing cell cultures has been instrumental in elucidating the diverse functions of sEVs within the CNS, particularly emphasizing the differences in sEV functions based on their cellular origin within the CNS. For instance, the release of sEVs from neurons and astrocytes is tightly regulated by depolarization events (Kaya et al., 2023; Rong et al., 2021). Extracted from culture supernatants, these vesicles carry a variety of cargoes, including prion proteins, cell adhesion molecules, and glutamate receptors, underscoring their integral role within neural networks. Beyond their roles in the CNS, sEVs are pivotal in modulating immune responses (Xu et al., 2020). For example, brain-derived sEVs can transport brain antigens into the peripheral circulation, influencing peripheral immune responses (Vaccari et al., 2016; Selmaj et al., 2017). Furthermore, sEVs can transmit inflammatory signals from peripheral immune tissues to the brain, cross the blood-brain barrier (BBB), and engage with innate immune cells (Fu et al., 2015; Li D. et al., 2020). This interaction initiates various immune responses, including the activation of macrophages, natural killer cells, and the maturation of dendritic cells, thereby enhancing antigen presentation (Chen and Chopp, 2018). Given their potential as diagnostic biomarkers for neurological diseases, the molecular content of sEVs secreted by brain cells is frequently analyzed (Goetzl et al., 2018). With their high specificity and minimal invasiveness, sEVs are emerging as promising tools for “liquid biopsies” (Jiang et al., 2020). These vesicles serve as natural carriers, facilitating efficient molecular transport due to their favorable biocompatibility (Duan et al., 2021). In the context of neurodegenerative diseases, sEVs have been implicated in the propagation of misfolded proteins along neuroanatomical pathways, functioning in a manner akin to prion-like mechanisms (Xiao et al., 2017). Prion infectivity and transmissibility arise from preformed aggregates that bypass the lag phase of aggregation, thereby accelerating the formation of additional aggregates (Puangmalai et al., 2020). Similarly, in neurodegenerative diseases, the uptake of pathogenic protein seeds by neurons induces the misfolding of native proteins, promoting the spread of these pathogenic proteins across different brain regions (Gomes et al., 2020). As a result, increasing attention is being directed toward understanding the pathogenesis of neurodegenerative disorders, with a particular focus on the mechanisms of pathogenic protein transmission and the role of sEVs derived from resident brain cells (Guitart et al., 2016). However, the precise cellular origins of the sEVs that contribute to the aggregation and dissemination of these pathogenic proteins remain uncertain (Guo et al., 2020). Moreover, sEVs play a critical role in clearing misfolded proteins, a process central to the pathogenesis of neurodegenerative diseases. Emerging therapeutic strategies involving mesenchymal stem cell-derived exosomes (MSC-exos) have shown promise in improving neurological outcomes in these conditions (Gorabi et al., 2019). MSC-exos are notably enriched with enzymes such as neprilysin and insulin-degrading enzyme, both of which are key to the clearance of pathological proteins (de Dios et al., 2019). This highlights the potential of MSC-exos as therapeutic agents in neurodegenerative diseases, offering a novel approach to combating protein misfolding and aggregation.

The intricate interplay between dysregulated microRNAs, oxidative stress, carboxyl-terminal fragments, and inflammation has garnered increasing attention in recent years. Emerging evidence underscores the pivotal role of neuroinflammation in the pathogenesis of AD (Ghosh et al., 2020; Lananna et al., 2020). Chronic exposure to an inflammatory microenvironment fosters sustained neuroinflammation, which ultimately leads to neuronal apoptosis. Within this framework, astrocytes and microglia are identified as the principal sources of proinflammatory mediators. Under normal physiological conditions, microglia are essential for maintaining brain homeostasis. However, in the presence of Aβ deposition, aging, and reactive oxygen species (ROS), microglia become hyperactivated, releasing proinflammatory cytokines such as interleukin-1α (IL-1α), interleukin-1β (IL-1β), tumor necrosis factor-α (TNF-α), and ROS (Liddelow et al., 2017; Leng and Edison, 2021; Cai et al., 2022). This activation initiates the recruitment and activation of neuroimmune cells, including astrocytes, which trigger innate immune responses, leading to a cascade of proinflammatory reactions (Heneka et al., 2015). Additionally, activated microglia stimulate the production of cathepsin B, exacerbating neuronal dysfunction (Chaney et al., 2019). Consequently, neuroinflammation accelerates AD pathology by promoting the accumulation of tau and Aβ proteins (Kinney et al., 2018). Elevated levels of proinflammatory factors and ROS have been observed around senile plaques, contributing to neuronal apoptosis (Ozben and Ozben, 2019). While activated astrocytes can exert neuroprotective effects by inhibiting Aβ aggregation, they also paradoxically promote the release of ROS and inflammatory cytokines, perpetuating the cycle of inflammation. Mesenchymal stem cells (MSCs) and, more specifically, MSC-derived EVs, have emerged as promising therapeutic strategies to counteract these pathological processes. MSC-derived EVs are enriched with lipids, proteins, enzymes, and microRNAs that possess anti-inflammatory properties, Aβ-degrading capabilities, and neurotrophic functions (Elia et al., 2019). For instance, the incubation of MSC-EVs with N2A neuroblastoma cells has been shown to reduce both secreted and intracellular Aβ peptide levels (Katsuda et al., 2013). Recent studies have further demonstrated that coculturing MSCs with rat neurons exposed to soluble Aβ oligomers (AβOs) leads to the internalization and degradation of AβOs, the release of EVs containing active catalase, and the selective secretion of interleukin-6, interleukin-10, and vascular endothelial growth factor (de Godoy et al., 2018). These findings suggest that MSCs protect against AD through three complementary mechanisms: (1) the internalization and degradation of AβOs, (2) the release of EVs containing active catalase, and (3) the selective secretion of anti-inflammatory cytokines and neurotrophic factors (Gatti et al., 2020). The potential of MSCs as a viable cell-based therapeutic strategy for AD, particularly in mitigating AβO-induced oxidative stress and synaptic damage, continues to be a major focus of ongoing research.

Extracellular vesicle (EV)-mediated neuroinflammation is increasingly recognized as a critical driver in the progression of AD, with mechanisms intricately linked to carboxyl-terminal fragments (CTFs) of amyloid precursor protein (APP). Aβ peptides are generated through the sequential cleavage of transmembrane APP by β- and γ-secretases (Checler, 1995). Initially, β-secretase cleaves APP to produce the membrane-bound fragment C99 (also known as βCTF), which is subsequently processed by γ-secretase, resulting in the release of soluble Aβ peptides. This proteolytic cascade defines the amyloidogenic pathway. Recent research has shown that C99, the direct precursor to Aβ, accumulates not only in AD-affected brains but also in AD-like mouse models. Interestingly, C99 accumulation occurs much earlier than Aβ deposition, suggesting that this metabolite may play a pivotal role in the early stages of AD pathology (Kaur et al., 2017; Cavanagh et al., 2013; Mondragón-Rodríguez et al., 2018). Inflammatory responses have been particularly pronounced in γ-secretase inhibitor-treated mice, which display minimal Aβ but elevated levels of APP-CTFs, reinforcing the link between C99 accumulation and neuroinflammation (Mondragón-Rodríguez et al., 2018; Lauritzen et al., 2019). Supporting this, Dr. Levy's team, using the APPE693Q mouse model, observed early-stage neuroinflammation preceding detectable Aβ, further connecting intraneuronal protein aggregates with the activation of neuroinflammatory processes (Mondragón-Rodríguez et al., 2018; Lauritzen et al., 2019). Moreover, EVs have been implicated in the accumulation of β-APP-CTFs in the brain, which are believed to initiate neurodegenerative processes associated with neuroinflammation (Kaur et al., 2017; Lauritzen et al., 2016). However, conflicting findings indicate that incubating EVs with γ-secretase inhibitors does not significantly alter APP-CTF levels, although a reduction in amyloid intracellular domain levels suggests that γ-secretase remains active within EVs (Pérez-González et al., 2020). This duality highlights the complex role of EVs: while they may provide neuroprotection by facilitating the secretion of APP and neurotoxic metabolites in the early stages of AD, they can also become sites for the generation and accumulation of APP metabolites in later stages, particularly when endosomal-lysosomal degradation systems are compromised (Pérez-González et al., 2020). Such accumulation may exacerbate AD-associated neurodegeneration. Therefore, elucidating the spatiotemporal roles of brain-derived EVs in AD is critical for advancing our understanding of AD pathogenesis.

In recent years, there has been increasing focus on investigating the relationship between microRNAs (miRNAs) and EVs in AD. MiRNAs have emerged as crucial regulators of gene expression, particularly in genes implicated in the pathogenesis of AD, including β-site APP-cleaving enzyme 1 (BACE-1), APP, tau, presenilin, and brain-derived neurotrophic factor (Ramakrishna and Muddashetty, 2019; Smith et al., 2015; Banzhaf-Strathmann et al., 2014; Keifer et al., 2015; Li et al., 2016). For instance, miR-9, miR-29a, miR-107, miR-125b, and miR-135b modulate BACE1 mRNA expression (Xie et al., 2017; Wang et al., 2008; Hébert et al., 2008; Li P. et al., 2020; Li R. et al., 2020; Zhang et al., 2016), while miR-106b and miR-132 suppress APP expression, influencing the progression of Aβ and tau pathologies (Zhang et al., 2016; Zhang and Bian, 2021). Additionally, other AD risk genes, such as glycogen synthase kinase-3 beta and neuron navigator 3, are regulated by miR-9-5p and miR-29a, respectively (Shioya et al., 2010; Liu et al., 2020). Notably, miR-132 targets glycosyltransferase-like domain-containing 1 and neuronal nitric oxide synthase, thereby promoting tau phosphorylation and apoptosis (Zhang and Bian, 2021). Furthermore, miR-135b targets Disrupted-in-Schizophrenia-1, a critical risk factor for neuropsychiatric disorders, as well as sirtuin 1, which has demonstrated neuroprotective effects in AD (Rossi et al., 2014; Li R. et al., 2020; Li et al., 2022). Given their significant role in AD pathogenesis, miRNAs have been studied as potential biomarkers for disease progression in brain tissue, CSF, and plasma. A meta-analysis of 10 studies involving 770 AD patients and 664 controls highlighted the diagnostic potential of circulatory miRNAs, demonstrating high sensitivity, specificity, and diagnostic odds ratios (Zhang et al., 2019). Similarly, a comprehensive review by Swarbrick et al. identified miR-125-5p and miR-132-3p in EVs as promising biomarkers for AD diagnosis, as these EV-associated miRNAs reflect the dysregulation observed in brain tissues (Li et al., 2023). Recent studies further revealed that miR-9-5p is consistently downregulated in both EVs and brain tissues of AD patients, and its therapeutic potential has been validated in murine and human cell models of AD (Li et al., 2023). Thus, miR-9-5p represents a promising candidate for the development of novel AD therapies.

### Therapeutic effects of EVs in AD

Figure 7B highlights the keyword “mesenchymal stem cells” in relation to the therapeutic potential of EVs in AD. Numerous studies utilizing transgenic mouse models have demonstrated the efficacy of mesenchymal stem cell-derived EVs (MSC-EVs) in addressing key pathological features of AD, such as amyloid plaques, neurofibrillary tangles, and neuroinflammation. These interventions have collectively led to enhanced neuronal protection, improved synaptic plasticity, and better cognitive outcomes (Rather et al., 2023). *In vitro* studies have also elucidated the molecular mechanisms underlying the effects of MSC-EVs, revealing their capacity to reduce Aβ levels and shift microglial phenotypes from pro-inflammatory to anti-inflammatory states (Rather et al., 2023). Emerging therapeutic strategies, including genetic modification, manipulation of the inflammatory microenvironment, and pretreatment of MSCs under hypoxia or 3D culture conditions, have been shown to further augment the neuroprotective properties of MSC-EVs (Rather et al., 2023). Additionally, recent findings suggest that intranasal administration of a peptide hydrogel can prolong the retention of MSC-EVs, thereby significantly enhancing their therapeutic efficacy in AD models (Huang M. et al., 2024). The immunomodulatory effects of MSCs are primarily mediated through their EVs, which participate in chronic inflammatory and immune processes by transferring nucleic acids, proteins, and lipids from parent cells to recipient cells. This mechanism allows MSC-EVs to retain their immunomodulatory functions while circumventing the safety concerns associated with live cell therapy, making them promising candidates for immunomodulatory treatments (Ye et al., 2023). Moreover, stem cell-derived EVs can be engineered to deliver therapeutic agents and modify surface properties, thereby enhancing their efficacy (Zhou et al., 2024). Despite these promising findings, further research is needed to elucidate the detailed mechanisms by which MSC-EVs exert therapeutic effects in AD. While many experimental results are encouraging, a deeper understanding of the intrinsic mechanisms, such as the specific targets and pathways by which EVs activate neurogenic niches, is essential. This research is crucial for advancing the clinical application of MSC-EVs. Although the neuroprotective effects of MSC-EVs have been primarily demonstrated *in vitro* and in animal models, it remains uncertain whether these benefits will translate effectively to human patients. Clinical trials are therefore necessary to verify these effects in human populations. Currently, several clinical trials are investigating the safety and efficacy of MSC-EVs in treating Alzheimer's disease. These trials are in the early stages, with some in Phase I and Phase II, focusing on evaluating the safety profile and potential therapeutic benefits of MSC-EVs in human patients. Additionally, refined translational models and tools are needed to bridge the gap between basic research and clinical practice, facilitating the successful implementation of EVs-based therapies. Addressing these challenges will significantly enhance the prospects of EVs in translational and regenerative medicine (Yuan Y. G. et al., 2023).

## Study limitations

This study has several limitations. First, only English-language papers from the WoS database were included, excluding publications in other languages. The Science Citation Index concept, based on Bradford's law in bibliometrics, defines a core set of journals considered world-leading due to their rigorous selection process. Therefore, while the publications included in WoS are generally representative of the discipline, they may not capture the full global research landscape. Second, papers published after the search date were not included, meaning that the growth trend of publications may extend beyond what our mathematical model predicted.

## Conclusion

Over the past 10–15 years, research on the role of EVs in AD has witnessed a marked increase, with a notable slow growth phase from 2001 to 2018 followed by a surge in research activity from 2018 to 2023. This period of acceleration has resulted in a total of 602 publications globally, reflecting the growing recognition of EVs as central players in AD pathogenesis. The United States and China currently lead in the number of publications in this field. The bibliometric analysis underscores that neuroinflammation, mediated by EVs, has become a central theme in the onset and progression of AD, supported by an expanding body of research examining the complex interactions between small EVs, oxidative stress, carboxyl-terminal fragments, microRNA dysregulation, and inflammation. While the precise role of neuroinflammation in AD pathogenesis and treatment remains debated, EVs, particularly those derived from MSC-EVs, have emerged as promising mediators of intercellular communication. MSC-EVs offer significant advantages such as low immunogenicity, reduced tumorigenic risk, and a favorable safety profile, positioning them as a novel and potentially safe nanotherapeutic approach for AD treatment. These findings highlight not only the rapid growth of research in this area but also the increasing recognition of MSC-EVs as multifunctional therapeutic candidates. Despite ongoing challenges, MSC-EVs hold considerable promise in advancing AD treatment strategies due to their unique biological functions. Future studies are crucial for further understanding the mechanisms underlying MSC-EVs' effects and their translation into clinical settings, particularly in human trials, to verify their efficacy and safety in treating AD.

## Data Availability

The original contributions presented in the study are included in the article/supplementary material, further inquiries can be directed to the corresponding author.
